# Neurologic outcome after out-of-hospital cardiac arrest could be predicted with the help of bispectral-index during early targeted temperature management

**DOI:** 10.1186/s13049-018-0529-7

**Published:** 2018-07-13

**Authors:** Jeong Ho Park, Jae Hun Oh, Seung Pill Choi, Jung Hee Wee

**Affiliations:** Department of Emergency Medicine, Yeouido St. Mary’s Hospital, The Catholic University of Korea, College of Medicine, 10, 63-ro, Yeongdeungpo-gu, Seoul, 07345 Republic of Korea

**Keywords:** Hypothermia, Induced, Prognosis, Out-of-hospital cardiac arrest

## Abstract

**Background:**

Outcome prediction is crucial for out-of-hospital cardiac arrest (OHCA) survivors. Several attempts have been made to use the bispectral index (BIS) for this purpose. We aimed to investigate the prognostic power of the BIS during the early stage of targeted temperature management (TTM) after OHCA.

**Methods:**

From Jan 2014 to Feb 2017, the BIS was determined in OHCA patients as soon as possible after the start of TTM. We injected a neuro-muscular blocking agent and recoded the BIS value and the time when the electromyographic (EMG) factor reached zero. The primary outcome was the cerebral performance category scale (CPC) score at 6 months, and a poor outcome was defined as a CPC score of 3, 4, or 5. The exclusion criteria were age under 18 years, traumatic cardiac arrest, and BIS data with a non-zero EMG factor.

**Results:**

Sixty-five patients were included in this study. Good outcomes were observed for 16 patients (24.6%), and poor outcomes were observed for 49 patients (75.4%). The mean time of BIS recording was 2.3 ± 1.0 h after return of spontaneous circulation (ROSC). The mean BIS values of the good outcome and poor outcome groups were 35.6 ± 13.1 and 5.5 ± 9.2, respectively (*p* < 0.001). The area under the curve was 0.961. Use of a cut-off value of 20.5 to predict a good outcome yielded a sensitivity of 87.5% and specificity of 93.9%. Use of a cut-off value of 10.5 to predict a poor outcome yielded a sensitivity of 87.8% and specificity of 100%.

**Conclusion:**

With the help of BIS, physicians could predict that a patient who has BIS value over 20.5 after ROSC could have a big chance to get good neurological outcome in less than three hours.

## Background

Sudden cardiac arrest leads to high mortality rates and lethal sequelae. To minimize these effects, many efforts have been made to develop an emergent medical service system and improve post-cardiac arrest care. Despite these efforts, the survival discharge rate and good neurologic outcome rate are 9.6 and 1.9%, respectively, in South Korea [1]. The survival discharge rate is similar to rates in North America (7.9–11.4%), Europe (10.7%), and Japan (12%). The percentage of cases in which the patients remain in a persistent coma after return of spontaneous circulation (ROSC) is reported to be approximately 11–12%; thus, this can be considered a serious social and economic problem [2].

In this sense, outcome prediction is one of the crucial components in post-cardiac arrest care. Outcome prediction could help decide the direction of treatment. For example, if the outcome prediction result is bad, the guardians of patients who have no chance of survival could be advised that further treatment should not be given because it is unnecessary. Because the withdrawal of life support is used in Europe when a bad prognosis is predicted, an accurate outcome prediction is critically important [3]. Several outcome prediction tools have been used, such as neurologic examination, neurophysiologic examination, biomarkers, and brain imaging. However, if prediction is performed only using any one of those tools, the accuracy will be low [4]. Thus, several attempts have been made to combine prediction tools.

The bispectral index (BIS) is a measure that indicates the extent of brain activity in numbers displayed on a screen by attaching a sensor to the forehead of the patient in a non-invasive manner. A BIS of 0 represents no brain activity, while a BIS of 100 represents complete wakefulness. In terms of reflecting the extent of brain activity of patients, many studies have been conducted on BIS monitoring as an outcome prediction method in a post-cardiac arrest setting to determine its ease of application and interpretation [5–8]. However, to date, the exact method, outcome prediction cut-off values and prediction timing have not been determined. Therefore, the authors aimed to investigate the speed and accuracy of the BIS, which was performed at the earliest possible stage of targeted temperature management (TTM), in predicting the neurologic outcome among comatose survivors after cardiac arrest to whom TTM had been applied.

## Methods

### Patients

This study was conducted on patients who had been treated with TTM and who were unable to obey commands after ROSC due to out-of-hospital cardiac arrest (OHCA). The patients’ core temperatures were maintained between 32 °C and 34 °C using surface cooling (ArcticGel™ pads, Artic Sun System® 5000, Medivance, Louisville, CO, USA) or an endovascular cooling system (ICY-catheter, CoolGard® 3000; Alsius, Irvine, CA, USA) for 24 h. Then, their core temperatures were increased to 36.5 °C at a rate pf 0.25 °C/hour. Patients with a history of neurological disease, a cerebral performance category (CPC) score less than 3 before cardiac arrest, traumatic cardiac arrest, and an age less than 18 years were excluded.

This study was prospectively conducted in the emergency department (ED) of Yeouido St. Mary’s Hospital, a teaching hospital in South Korea. The study was approved by the Institutional Review Board of the Catholic University of Korea, Yeouido St. Mary’s Hospital. The study period was from January 2014 to February 2017. The primary outcome was the CPC score at six months after ROSC. CPC scores of 1 and 2 corresponded to a good outcome, whereas CPC scores of 3–5 corresponded to a poor outcome. Neurological status at 6 months of ROSC was assessed by telephone interview after survival discharge. The CPC was assessed by two of the researchers, and the BIS values were blinded at that time.

### BIS monitoring method

BIS (Aspect medical systems®, Inc. Netherlands) monitoring was started when the patient was determined to require TTM after ROSC, and TTM treatment was continued until the patient’s temperature recovered to a normal value after rewarming. The hospital began TTM in the ED and continued after transfer to the intensive care unit (ICU). The BIS value and time of measurement were recorded and analysed when the patient’s electromyographic (EMG) component reached zero. A neuro-muscular (NM) blocking agent was injected as a bolus after the start of TTM during the removal of the EMG component. The BIS value was considered significant only when the signal quality indicator (SQI) was 80% or higher.

### Statistical analysis

The statistical analysis was performed with PASW statistics ver. 18.0 (SPSS Inc., Chicago, USA). Normality was tested using the Kolmogorov-Smirnov test, and categorical variables are expressed as frequencies and percentages. Chi-squared or Fisher’s exact tests were used to compare the two groups. In cases of normal distributions, the mean values were compared using a t-test, while in cases of non-normal distributions, the median values were compared using the Mann-Whitney test. Receiver operating characteristic (ROC) curve was constructed with reference line to determine the mean cut-off value of the initial BIS value with the highest sensitivity and specificity for prediction of good neurological outcomes. A *P*-value of less than 0.05 was considered statistically significant.

## Results

**Fig. 1:**
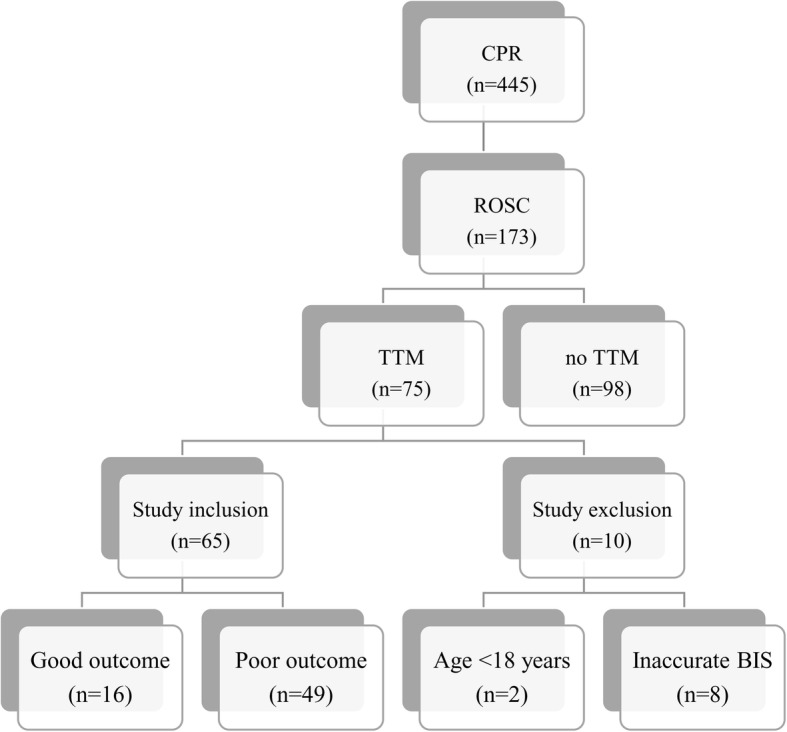
Diagram of patients study inclusion. CPR, cardiopulmonary resuscitation; ROSC, return of spontaneous circulation; TTM, targeted temperature management; BIS, bispectral index

During the study period, OHCA occurred in 445 patients, ROSC was achieved in 173 patients, and TTM treatment was performed in 75 patients. Among these patients, 2 patients were younger than 18 years old, and 8 patients’ BIS data were inaccurate, thus 10 were excluded from this study. Consequently, 65 patients were included in this study (Fig. 1). Seventy-five percent of patients were males (*N* = 49), and their average age was 55.6 ± 16.8 years old. Witnessed arrest occurred in 63.1% of patients (*N* = 41). A non-shockable initial rhythm was found in 75.4% of patients (N = 49), while a shockable rhythm was found in 23.1% of patients (*N* = 15), and one was difficult to identify the initial rhythm. The most frequent cause of cardiac arrest was cardiogenic aetiology (56.9% of patients, *N* = 37). The average time from arrest to ROSC was 31.6 ± 16.9 min. At six months after ROSC, 24.6% of patients had good outcomes (*N* = 16), while 75.4% had poor outcomes (*N* = 49) (Table 1).

**Table 1:** Baseline demographics and clinical characteristics of victims

Variables	*N* = 65
Male, *n* (%)	49 (75.4)
Age, years	55.6 ± 16.8
Witness arrest, *n* (%)	41 (63.1)
Initial rhythm, *n* (%)	
Non-shockable	49 (75.4)
Shockable	15 (23.1)
Unknown	1 (1.5)
Cause of cardiac arrest, *n* (%)	
Cardiogenic	37 (56.9)
Respiratory	14 (21.5)
Hanging	5 (7.7)
Drowning	7 (10.8)
Drug	2 (3.1)
Time from arrest to ROSC, min	31.6 ± 16.9
Time from ROSC to TTM start, hr	1.6 ± 0.5
Time from ROSC to initial BIS monitoring, hr	2.3 ± 1.0
Body temperature at BIS monitoring, °C	35.2 ± 1.3
CPC 1–2, *n* (%)	16 (24.6)
CPC 3–5, *n* (%)	49 (75.4)

*ROSC* return of spontaneous circulation, *TTM* targeted temperature management, *BIS* Bispectral index, *CPC* cerebral performance category

**Fig. 2:**
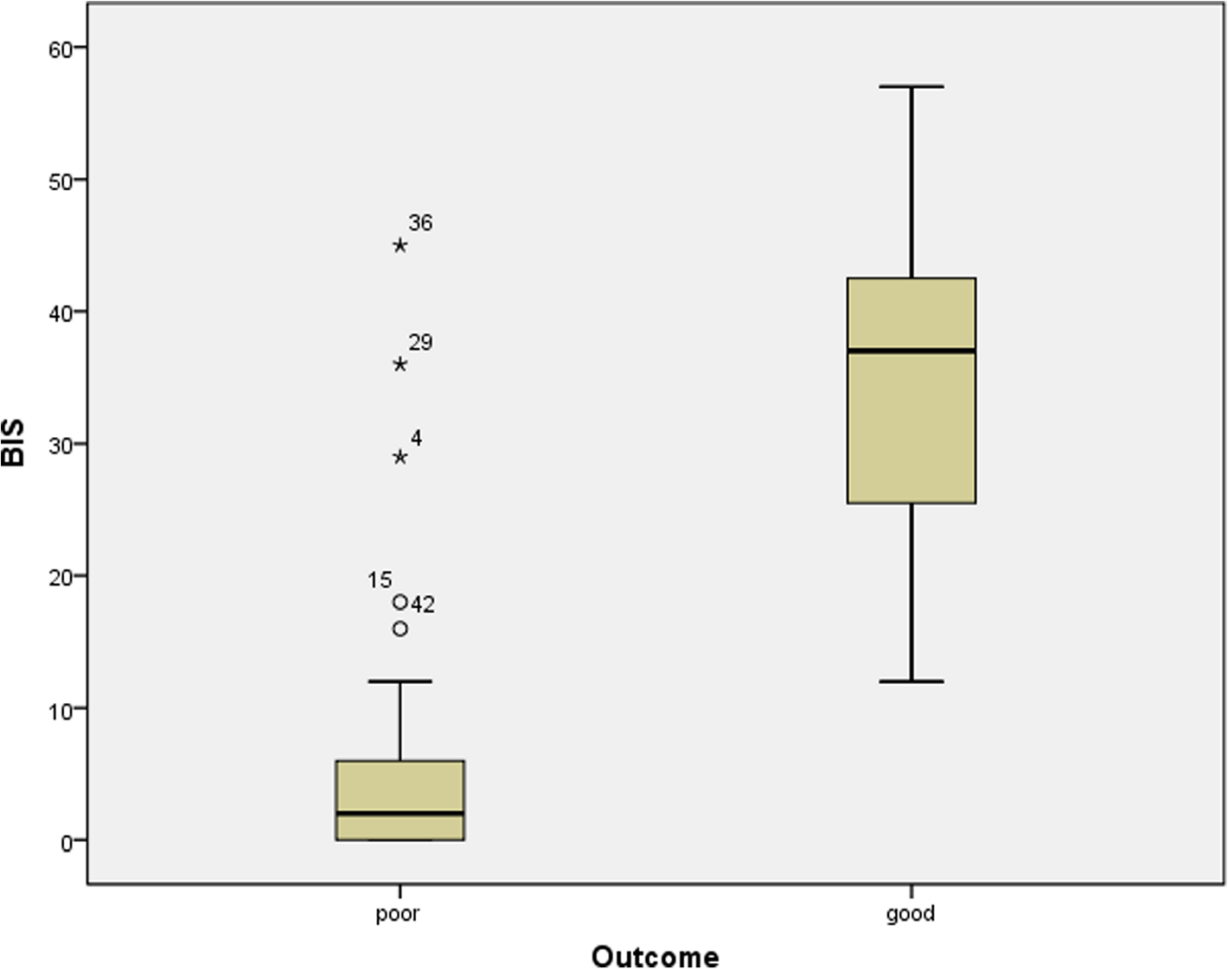
Box plots of bispectral index (BIS) in good outcome and poor outcome. Data are presented as median, quartile 1, quartile 3, and outliers. The outliers were included in the mean BIS analysis

**Fig. 3:**
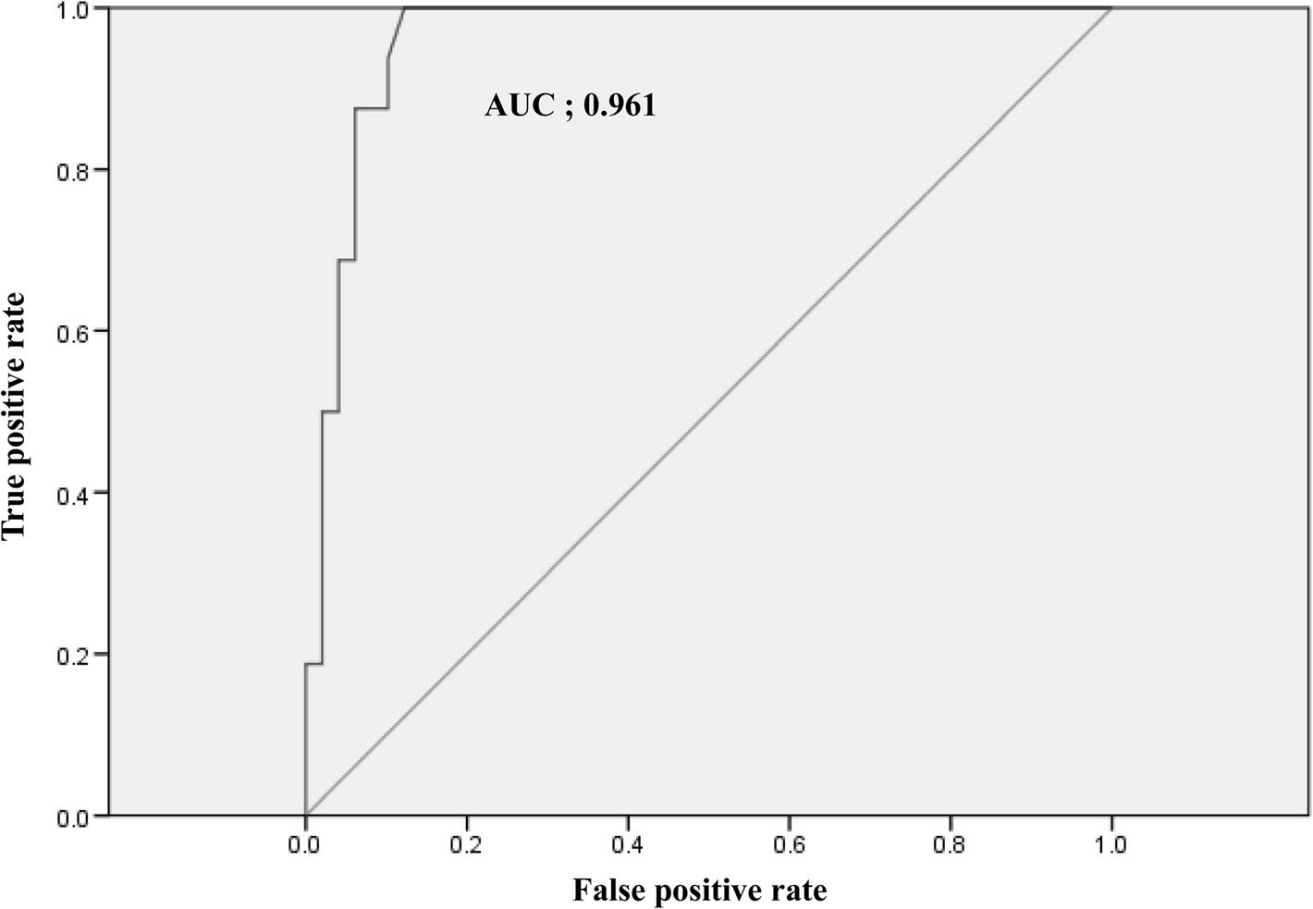
Receiver operating characteristic curves for bispectal index outcome prediction. The area under the curve is 0.961. AUC; area under the curve

The average time from ROSC until the first significant BIS value was obtained was 2.3 ± 1.0 h. The mean BIS value for the good outcome group was 35.6 ± 13.1, which was significantly higher than the mean BIS value for the poor outcome group (5.5 ± 9.2, *P* < 0.001) (Fig. 2). Among poor outcome patients, 17 patients showed zero BIS values. And 15 patients died within 72 h after ROSC and their mean BIS value was 2.4 ± 3.0. The ROC curves used to determine the ability of the BIS to predict a good outcome are shown in Fig. 3. The area under the curve (AUC) was 0.961 (95% confidence interval, 0.910–1.0, *P* < 0.001). A cut-off value ≥20.5 could predict a good outcome with 87.5% sensitivity and 93.9% specificity. A cut-off value < 10.5 could predict a poor outcome with 87.8% sensitivity and 100% specificity. (Table 2).

**Table 2:** Sensitivity, specificity, positive, negative predictive value, and accuracy for good or poor outcome prediction according to BIS cutoff value

BIS Cut-off value		Sensitivity (95% CI)	Specificity (95% CI)	PPV (95% CI)	NPV (95% CI)	Accuracy (95% CI)
For good	> 20.5	87.5 (61.7–98.5)	93.9 (83.1–98.7)	82.4 (60.6–93.4)	95.8 (86.3–98.8)	92.3 (83.0–97.5)
For poor	< 10.5	87.8 (75.2–95.4)	100 (79.4–100)	100	72.7 (55.8–85.0)	90.8 (81.0–96.5)

*BIS* bispectral index, *PPV* positive predictive value, *NPV* negative predictive value, *CI* confidence interval

## Discussion

The studies conducted to date that have explored the relationship between the BIS value and neurologic outcomes in cardiac arrest survivors have mostly focused on observing BIS trends for patients receiving TTM in the ICU and identifying the most suitable timing and value for outcome prediction. However, this study shifted the focus to understanding whether the BIS value measured at the earliest time after the start of TTM would be helpful for predicting patient outcomes. In the 2015 American Heart Association Guidelines Update for Cardiopulmonary Resuscitation and Emergency Cardiovascular Care, in comatose adult patients with ROSC after OHCA, TTM treatment is recommended for Class I, whether whose cardiac arrest rhythm is shockable or not, and TTM is also recommended as class I in in-hospital cardiac arrest [9]. Despite this, many survivors suffer from sequelae due to hypoxic brain injury. According to a multicentre study in South Korea, 556 (59.8%) of 930 patients who received TTM were discharged alive, and 249 patients (26.8%) obtained good neurological outcomes. Hence, 307 patients who were discharged alive showed poor neurological outcomes [10]. Therefore, withdrawal of life sustaining therapy is performed for post-cardiac arrest patients showing a poor neurologic prognosis in some regions in Europe and America [11]. Thus, for cardiac arrest patients, outcome prediction is as critically important as treatment with TTM. Furthermore, outcome prediction should be performed as early as possible; however, the outcome should be accurate. The tool used for assessment should be easy to apply and result in no difference in interpretation between physicians. The neurological examination, such as pupillary light reflexes, corneal reflexes, and motor response is good to predict poor outcome, but there is a disadvantage that interpretation between testers may be different. Electroencephalogram is widely used to assess brain function, but it is expensive to examine and expert help is needed to interpret the results. Somatosensory evoked potentials recording requires appropriate skills and experience to apply. Blood markers such as NSE and S-100 have the possibility of high false positive rate, they should not be used alone to predict neurologic outcome [9]. In these aspects, BIS has several advantages. The BIS is not susceptible to interpretational differences because it presents brain function using values ranging from 0 to 100. The numerical representation also has another advantage in that professional knowledge and skill are not required to interpret the meaning of the value. Additionally, it is simple to attach an electrode on the patient’s forehead. Thus, the BIS may be a suitable tool for prognostic prediction [12].

Numerous studies have been conducted on BIS measurement timing for neurologic outcome prediction among OHCA patients. Chollet-Xemard et al. stated that the BIS is not applicable for prognostic prediction if it is applied during cardio-pulmonary resuscitation (CPR) [13]. BIS values are helpful for prognostic prediction among OHCA patients, but the timing has varied in different studies, e.g., one study reported that time was 4 h after CPR, another reported 267 min after CPR, and another reported 12.5 h after ROSC [5, 6, 14]. The researchers in this study attempted to record the BIS as early as possible after the start of TTM. By injecting an NM blocker, the measured BIS value reached a stabilized state after the EMG component reached “zero”, and therefore the BIS value represents only the brain function of patients. The reason for using this method was that a higher BIS value may be measured due to muscle movements, including self-respiration, shivering and seizure movement [15]. Complying with this protocol, this study found that the BIS value measured at a mean of 2.3 h after ROSC was helpful for outcome prediction of patients. This value was obtained an earlier time than suggested by other studies. This measurement could be obtained earlier than in other studies because TTM was started when the patient was in the ED. This early application of TTM contrasts with the fact that TTM was applied after patients were moved to ICU in other studies.

If the first BIS value obtained after the start of TTM and NM blocker injection is 20.5 or higher, a good neurological outcome can be predicted with 87.5% sensitivity and 93.9% specificity. Many patients who experience cardiac arrest and achieve ROSC are likely to have an unstable haemodynamic state due to myocardial stunning [16]. At this point, a high initial BIS value might indicate that hypoxic brain injury is not serious. Therefore, if physicians attempt to stabilize the haemodynamic state of a patient with cardiovascular dysfunction in an aggressive manner, a good result can be anticipated in terms of survival and neurological outcome. In contrast, if the BIS value is less than 10, a poor outcome could be predicted with 87.8% sensitivity and 100% specificity. This value is higher than that of Pascal et al., who predicted a poor outcome with 100% specificity when the BIS value was less than 2.4 at 271 min [6]. Among 49 patients who showed poor outcomes, the number of patients with a BIS value of 0 was 17. This result is consistent with that of Ward et al., who suggested that a poor outcome resulted with a BIS value of “zero” [17]. However, it should be noted that a lower BIS value could be measured due to the patient’s condition, such as a low haemodynamic state, hypothermia, and hypoglycaemia [18]. Hence, great care must be taken in performing outcome prediction for cardiac arrest survivors.

We proposed a cutoff value of 20.5 for good outcome prediction and 10.5 for poor outcome prediction. Leary et al. reported a cutoff point of 45 for the BIS value of 24 h after ROSC to predict good outcome, and Eertmans et al. reported that BIS value lower than 25 at 12 h of ROSC was predictive of poor outcome [19, 20]. Value of 45 and 25 are higher than our values, those may be because they did not adjust EMG value to zero in study. Also, Eertmans et al. concluded that that there was no correlation between EMG and BIS when the BIS value was lower than 25. However, further studies are needed to determine whether there is an effect of EMG on the BIS predictive power.

This study has several limitations. First, it is a single centre study with a relatively small sample size of 65. In future studies, it will be necessary to extend the study period and increase the sample size by conducting a multicentre study and applying the same BIS protocol. Second, the patient’s seizure movement might not be detected externally due to injection of NM blockers at the initial stage in this study. One recent study reported that a sudden increase in the BIS value and greater fluctuation are highly associated with seizure [21]. Although the BIS incorporates raw EEG data, only the BIS value was observed and considered in this study, which might not accurately represent the patient’s brain function if seizure is noted on the EEG. Therefore, it would also be important to identify and consider the correlation between these factors by performing BIS value monitoring and applying continuous EEG monitoring. Third, eight patients (10.9%) were excluded from this study because the SQI was less than 80. This implies that BIS may not be able to measure prognosis in approximately 10% of patients. Fourth, the researchers were not blinded to the BIS value and patients’ clinical outcomes. Therefore, the possibility that the researchers in this study performed treatment with a biased opinion due to being influenced by the BIS value cannot be excluded.

## Conclusion

Early (2.3 h) BIS measurement when the EMG component reaches “zero” and the SQI exceeds 80 after the start of TTM could predict the neurological outcome of patients after cardiac arrest. According to our results, a BIS value greater than 20.5 is associated with good outcomes with 87.5% sensitivity and 93.9% specificity. So we could predict person who have over 20.5 BIS value within 3 h after ROSC could have a big chance to get good neurological outcome. Further large and prospective investigations of the association between the BIS and neurological outcome will be required to establish an accurate method to determine the appropriate timing and values for BIS monitoring.

## Abbreviations

AUC: Area under the curve
BIS: Bispectral index
CPC: A cerebral performance category
CPR: Cardio-pulmonary resuscitation
ED: Emergency department
EMG: Electromyographic
ICU: Intensive care unit
OHCA: Out-of-hospital cardiac arrest
ROC: Receiver operating characteristic
ROSC: Return of spontaneous circulation;
SQI: Signal quality indicator
TTM: Targeted temperature management
